# Transcriptome and Metabolome Analyses of the Flowers and Leaves of *Chrysanthemum dichrum*

**DOI:** 10.3389/fgene.2021.716163

**Published:** 2021-08-31

**Authors:** Hua Liu, Xiaoxi Chen, Hạixia Chen, Jie Lu, Dongliang Chen, Chang Luo, Xi Cheng, Yin Jia, Conglin Huang

**Affiliations:** ^1^Beijing Key Laboratory of Agricultural Genetic Resources and Biotechnology, Beijing Agro-Biotechnology Research Center, Beijing Academy of Agriculture and Forestry Sciences, Beijing Engineering Research Center of Functional Floriculture, Beijing, China; ^2^Sichuan Agricultural University, College of Landscape Architecture, Chengdu, China; ^3^Beijing University of Agriculture, College of Landscape Architecture, Beijing, China; ^4^Shandong Forestry Protection and Development Service Center, Jinan, China

**Keywords:** *Chrysanthemum dichrum*, transcriptome, RNA-Seq, metabolites, metabolomics

## Abstract

*Chrysanthemum dichrum* is an important wild species in the family Asteraceae. However, because of a lack of genetic information, there has been relatively little research conducted on the molecular mechanisms in *C. dichrum*. There is no report describing the transcriptome and metabolome of *C. dichrum* flowers and leaves at different developmental stages. In this study, high-throughput sequencing and RNA-seq analyses were used to investigate the transcriptome of *C. dichrum* leaves, flower buds, and blooming flowers. Additionally, these three tissues also underwent a metabolomics analysis. A total of 447,313,764 clean reads were assembled into 77,683 unigenes, with an average length of 839 bp. Of the 44,204 annotated unigenes, 42,189, 28,531, 23,420, and 17,599 were annotated using the Nr, Swiss-Prot, KOG, and KEGG databases, respectively. Furthermore, 31,848 differentially expressed genes (DEGs) were detected between the leaves and flower buds, whereas 23,197 DEGs were detected between the leaves and blooming flowers, and 11,240 DEGs were detected between the flower buds and blooming flowers. Finally, a quantitative real-time Polymerase Chain Reaction (qRT-PCR) assay was conducted to validate the identified DEGs. The metabolome data revealed several abundant metabolites in *C. dichrum* leaves, flower buds, and blooming flowers, including raffinose, 1-kestose, asparagine, glutamine, and other medicinal compounds. The expression patterns of significant DEGs revealed by the transcriptome analysis as well as the data for the differentially abundant metabolites in three *C. dichrum* tissues provide important genetic and metabolic information relevant for future investigations of the molecular mechanisms in *C. dichrum*. Moreover, the results of this study may be useful for the molecular breeding, development, and application of *C. dichrum* resources.

## Introduction

*Chrysanthemum dichrum*, which is a perennial herb belonging to the genus *Chrysanthemum* in the family Asteraceae, is a very important germplasm resource. It is a wild relative of the famous ornamental plant *Chrysanthemum morifolium*. A *C. dichrum* plant can be more than 30 cm tall. Its main stem, which lies prone or is slanted, is bare, brown, and has multiple branches on the upper part, which contains densely appressed pubescent leaves. It is a highly edible plant with medicinal value and is used for landscaping as a garden greening material. *Chrysanthemum dichrum* is distributed in southwestern Hebei province in China (Hsianshiu, 1993). However, it can be introduced to other regions relatively easily, making it a wild flower species potentially useful as a functional flower crop.

Current research on *C. dichrum* mainly focuses on cross breeding and stress resistance, including tolerance to cold and drought. Previous studies proved that *C. dichrum*, *Chrysanthemum indicum* “Nankingense,” and other interspecific hybrid progeny are highly tolerant to drought conditions (Li et al., 2020). Chi et al. in order to solve the economic benefit and ornamental quality decrease caused by low temperature in northern China. The genetic control mechanism of interspecific cold tolerance between *C. dichrum* and *C. nankingense* was clarified by analyzing the cold-resistant traits such as Low Semi-Lethal Temperature (LT50), basal shoots height and number in the basal shoots stage of the hybrids (Chi et al., 2018). A research team found a 1,474 bp stress-induced CdDREBa promoter in *C. dichrum*. Identified a stress-inducible CdDREBa promoter from *C. dichrum*, Clothes several candidate stress-related *cis-*acting elements (MYC-box, MYB site, GT-1, And W-box) within it. CdDREBa promoter features a strong low temperature- and right-inducible promoter. This provides an attractive target for engineering inducible promoters in transgenic crops (Chen Y. et al., 2012). Additionally, the *CdICE1* gene in *C. dichrum* was cloned and an analysis of its cold tolerance-related function in *Arabidopsis thaliana* revealed that the miR398-CSD pathway is involved in the induction of freezing resistance (Chen Y. et al., 2013). Earlier research also confirmed that the structural expression of *ICE1* gene in *C. dichrum* in large Chrysanthemum improves the tolerance to low temperature, salt and drought. *CdICE1* represents a promising candidate for a biotechnological approach to improve the level of crop abiotic stress tolerance (Chen L. et al., 2012). Other studies applied RNA-seq technology to investigate chrysanthemum and related species (Chen et al., 2009; Wang et al., 2013; Xu et al., 2013; Ren et al., 2014; Hong et al., 2015; Liu et al., 2015), but the *C. dichrum* transcriptome and metabolome have been rarely examined. Accordingly, research regarding the *C. dichrum* transcriptome may increase our understanding of the gene regulatory mechanism and biological pathways in this wild species, while also identifying the downstream target genes of key transcription factors to clarify specific processes (Liu et al., 2013). Therefore, in this study, we analyzed the transcriptome and metabolome of *C. dichrum* leaves, flower buds, and blooming flowers. Moreover, we identified the genes differentially expressed in the examined plant tissues. We also determined the metabolite composition and content in the leaves, flower buds, and blooming flowers and identified some highly abundant economically valuable metabolites with substantial developmental potential. The findings of this study lay the foundation for future investigations regarding chrysanthemum resource development as well as molecular research and breeding related to *C. dichrum*.

## Results

### Illumina Sequencing and Assembly

**FIGURE 1:**
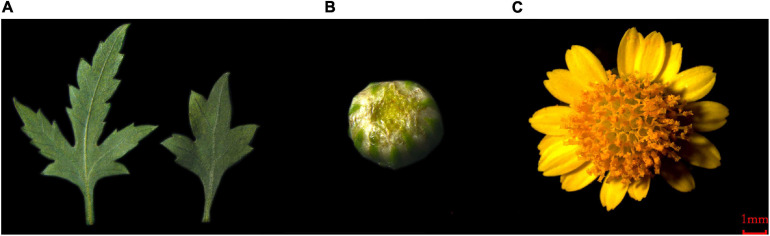
The plant material of *C. dichrum*. **(A)** *C. dichrum* leaves. **(B)** *C. dichrum* flower bud. **(C)** *C. dichrum* blooming flower.

This study involved analyses of *C. dichrum* leaves, flower buds, and blooming flowers (Figure 1). The transcriptomes of these plant tissues were sequenced using the Illumina HiSeq^TM^ 4000 platform. A total of 445,396,894 clean reads were obtained and 77,683 unigene sequences were assembled using *de novo* assembly technology. The N50 was 1,413 bp and the maximum and minimum lengths were 14,459 and 201 bp, respectively, with an average length of 839 bp (Table 1).

**TABLE 1:** *De novo* sequence assembly results.

Genes Num	GC%	N50 (bp)	Max length (bp)	Min length (bp)	Average length (bp)	Total assembled bases
77683	39.4979	1,413	14,459	201	839	65,184,963

### Gene Annotation and Functional Classification

**FIGURE 2:**
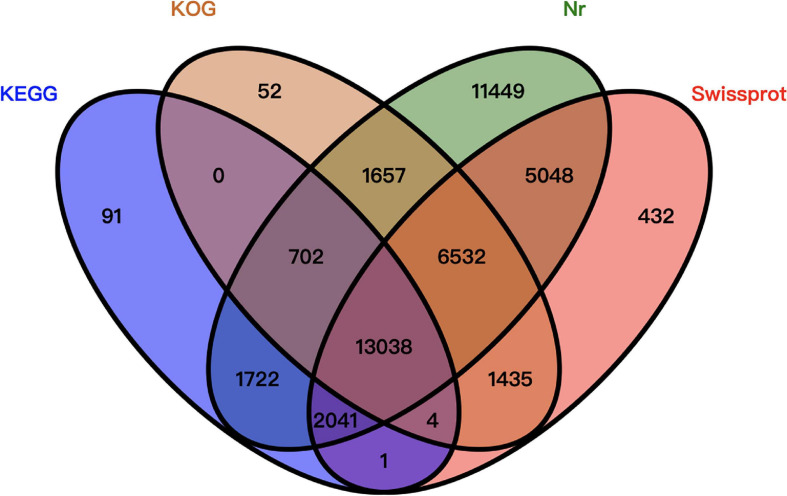
Venn diagram summarizing the unigene annotations based on four databases.

Of the 77,683 unigenes, 44,204 were annotated. More specifically, 42,189 unigenes were annotated using the Nr database, 28,531 unigenes were annotated using the Swiss-Prot database, 23,420 unigenes were annotated using the KOG database, and 17,599 unigenes were annotated using the KEGG database. A total of 33,479 unigenes were not been annotated (Figure 2).

**FIGURE 3:**
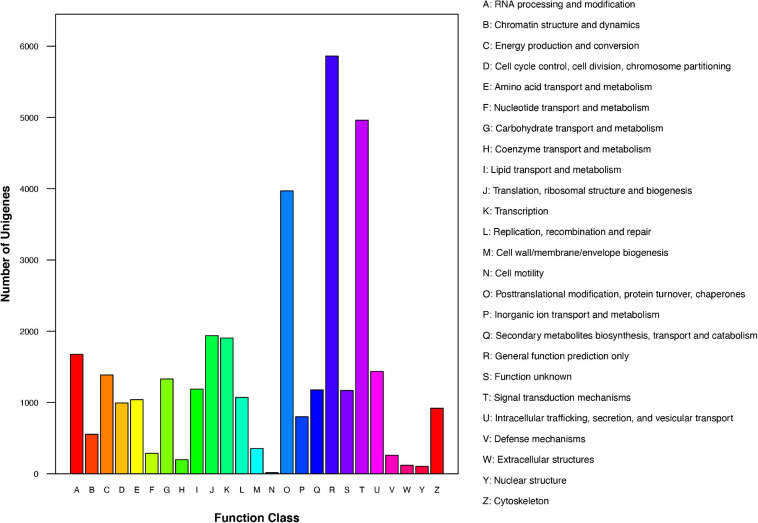
KOG functional classification of *Chrysanthemum dichrum* unigenes.

We further analyzed the annotated genes regarding their KOG classifications. As indicated in Figure 3, of the 25 KOG database functional categories, “General function prediction only” (5,862 unigenes), “Signal transduction mechanisms” (4,961 unigenes), and “Posttranslational modification, protein turnover, chaperones” (3,970 unigenes) had the most unigenes. In contrast, “Extracellular structures” (119 unigenes), “Nuclear structure” (105 unigenes), and “Cell motility” (16 unigenes) had the fewest unigenes.

**FIGURE 4:**
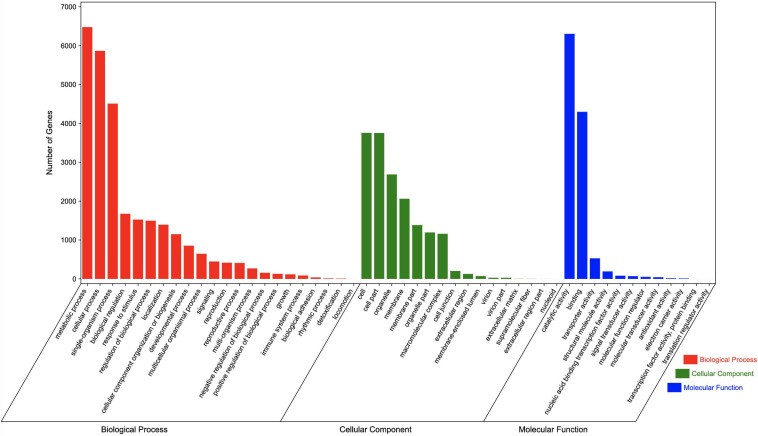
GO terms assigned to *Chrysanthemum dichrum* unigenes.

Regarding the GO analysis, 55,748 unigenes were classified into 50 groups in the three main categories. The biological process category had the most unigenes (27,682). “Metabolic process” and “Cellular process” were the main GO terms in this category, with 6,476 and 5,866 unigenes, respectively. A total of 16,466 unigenes were classified in the cell component category, with most annotated with the “Cells” (3,756 unigenes) and “Cell parts” (3,754 unigenes) GO terms. The molecular function category included 11,599 annotated unigenes, with “Catalytic activity” (6,303 unigenes) and “Binding” (4,296 unigenes) representing the main terms. Additionally, of the GO terms in the biological process, cell component, and molecular function categories, “Locomotion,” “Nucleoid,” and “Translation regulator activity” were assigned to the fewest genes, respectively (Figure 4).

The enriched KEGG pathways among the annotated unigenes were also determined. A total of 7,696 unigenes were associated with 134 pathways (Table 2), with “Metabolic pathways” assigned the most unigenes (3,480, 38.66%), followed by “Biosynthesis of secondary metabolites” (1,914, 21.26%). Notably, “Glycosphingolipid biosynthesis-lacto and neolacto series” (2, 0.02%) and “Anthocyanin biosynthesis” (1, 0.01%) were assigned the fewest unigenes.

**TABLE 2:** Enriched KEGG pathways among *Chrysanthemum dichrum* unigenes.

KEGG Categories	Unigene number	Pathway ID
Metabolic pathways	3480 (38.66%)	ko01100
Biosynthesis of secondary metabolites	1914 (21.26%)	ko01110
Ribosome	778 (8.64%)	ko03010
Carbon metabolism	570 (6.33%)	ko01200
Plant-pathogen interaction	503 (5.59%)	ko04626
Protein processing in endoplasmic reticulum	459 (5.1%)	ko04141
Biosynthesis of amino acids	444 (4.93%)	ko01230
Plant hormone signal transduction	411 (4.57%)	ko04075
Spliceosome	397 (4.41%)	ko03040
Endocytosis	360 (4%)	ko04144
RNA transport	320 (3.55%)	ko03013
Oxidative phosphorylation	285 (3.17%)	ko00190
Purine metabolism	285 (3.17%)	ko00230
Glycolysis / Gluconeogenesis	275 (3.05%)	ko00010
Ubiquitin mediated proteolysis	267 (2.97%)	ko04120
MAPK signaling pathway–plant	227 (2.52%)	ko04016
Starch and sucrose metabolism	224 (2.49%)	ko00500
mRNA surveillance pathway	224 (2.49%)	ko03015
Pyrimidine metabolism	213 (2.37%)	ko00240
Aminoacyl-tRNA biosynthesis	213 (2.37%)	ko00970
Phenylpropanoid biosynthesis	211 (2.34%)	ko00940
RNA degradation	202 (2.24%)	ko03018
Pyruvate metabolism	200 (2.22%)	ko00620
Amino sugar and nucleotide sugar metabolism	187 (2.08%)	ko00520
Phagosome	185 (2.06%)	ko04145
Carbon fixation in photosynthetic organisms	181 (2.01%)	ko00710
Cysteine and methionine metabolism	168 (1.87%)	ko00270
Ribosome biogenesis in eukaryotes	168 (1.87%)	ko03008
Glutathione metabolism	166 (1.84%)	ko00480
Glycerophospholipid metabolism	164 (1.82%)	ko00564
Glyoxylate and dicarboxylate metabolism	164 (1.82%)	ko00630
Fatty acid metabolism	163 (1.81%)	ko01212
Nucleotide excision repair	163 (1.81%)	ko03420
Homologous recombination	162 (1.8%)	ko03440
Peroxisome	161 (1.79%)	ko04146

### Analysis of Differentially Expressed Genes in *Chrysanthemum dichrum*

**FIGURE 5:**
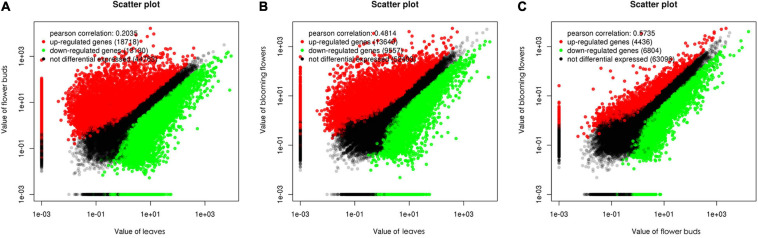
Scatter plots of differentially expressed genes among three *Chrysanthemum dichrum* plant tissues. The values in the vertical and vertical coordinates are gene expression levels. **(A)** The x-coordinate is the gene expression level of leaves, and the y-coordinate is the gene expression level of flower buds. **(B)** The x-coordinate is the expression level of leaves, and the y-coordinate is the expression level of blooming flowers. **(C)** The abscissa is the gene expression level of flower buds, and the ordinate is the gene expression level of blooming flowers.

We used a false discovery rate (FDR) < 0.05 and a |log_2_(fold-change)| > 1 as the thresholds for identifying differentially expressed genes (DEGs). A total of 31,848 genes were differentially expressed between the *C. dichrum* leaves and flower buds. Compared with the leaf expression levels, 18,718 and 13,130 DEGs had significantly up-regulated and down-regulated expression levels in the flower buds. A total of 23,197 DEGs were identified between the leaves and the blooming flowers. Compared with the corresponding expression during the blooming period, 13,640 and 9,557 DEGs had significantly up-regulated and down-regulated expression levels in the leaves. A comparison between the flower buds and blooming flowers revealed 11,240 DEGs, of which 4,436 and 6,804 DEGs had significantly up-regulated and down-regulated expression levels in the flower buds (Figure 5).

**FIGURE 6:**
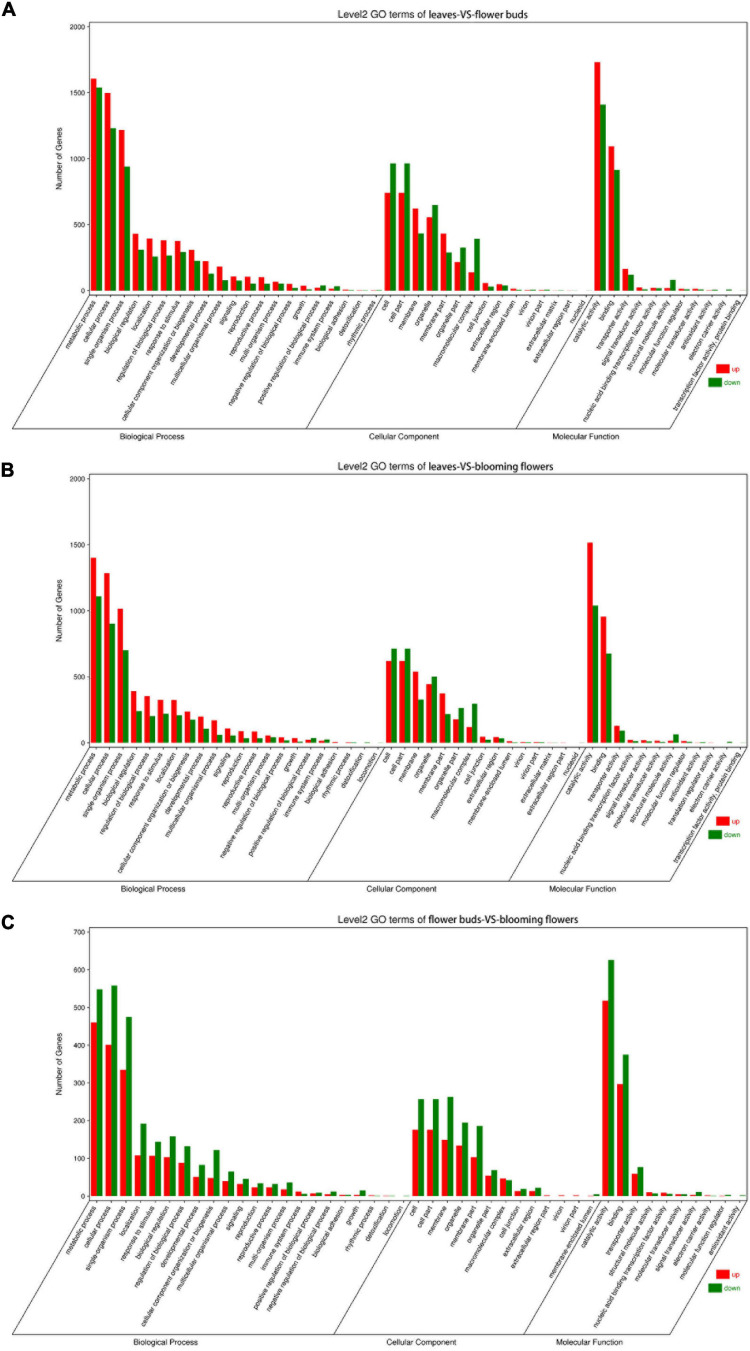
GO term classification of differentially expressed genes among *Chrysanthemum dichrum* plant tissues. The GO classification of all genes was divided into three parts: Biological Process, Cellular Component and Molecular Function. **(A)** Shows the GO classification of the differentially expressed genes in the leaves and flower buds comparison group, and **(B)** shows the GO classification of the differentially expressed genes in the leaves and blooming flowers comparison group. **(C)** Shows the GO classification of differentially expressed genes between the flower buds and blooming flowers comparison group.

The DEGs underwent GO and KEGG pathway enrichment analyses to determine the differences in biological processes and pathways among the leaves, flower buds, and blooming flowers. The DEGs between the leaves and flower buds were annotated with 47 GO terms. In the biological process category, the main GO terms were “Metabolic process” (GO:0008152), “Cellular process” (GO:0009987), and “Single-organism process” (GO:0044699), which were assigned to 3,146, 2,729, and 940 DEGs, respectively. In the cellular component category, the most represented GO terms were “Cell” (GO:0005623), “Cell part” (GO:0044464), and “Organelle” (GO:0043226), which were assigned to 1,706, 1,706, and 1,056 DEGs, respectively. The most common molecular function GO terms among the DEGs were “Catalytic activity” (GO:0003824), “Binding” (GO:0005488), and “Transporter activity” (GO:0005215), which were assigned to 3,142, 2,008, and 284 DEGs, respectively. A total of 134 KEGG pathways were enriched among 4,253 DEGs. The main enriched KEGG pathways were “Metabolic pathways” (ko01100), “Biosynthesis of secondary metabolites” (ko01110), and “Ribosome” (ko03010), which were associated with 1,853, 1,063, and 364 DEGs, respectively (Figure 6A).

The DEGs between the leaves and blooming flowers were annotated with 46 GO terms. In the biological process category, the main GO terms were “Metabolic process” (GO:0008152), “Cellular process” (GO:0009987), and “Single-organism process” (GO:0044699), which were assigned to 2,511, 2,187, and 1,716 DEGs, respectively. In the cellular component category, the most represented GO terms were “Cell” (GO:0005623), “Cell part” (GO:0044464), and “Membrane” (GO:0016020), which were assigned to 1,333, 1,333, and 867 DEGs, respectively. The most common GO terms in the molecular function category were “Catalytic activity” (GO:0003824), “Binding” (GO:0005488), and “Transporter activity” (GO:0005215), which were assigned to 2,555, 1,631, and 221 DEGs, respectively. A total of 132 KEGG pathways were enriched among 3,373 DEGs. The main pathways were “Metabolic pathways” (ko01100), “Biosynthesis of secondary metabolites” (ko01110), and “Ribosome” (ko03010), which were associated with 1,477, 843, and 270 DEGs, respectively (Figure 6B).

The DEGs between the flower buds and blooming flowers were annotated with 45 GO terms. In the biological process category, the main GO terms were “Metabolic process” (GO:0008152), “Cellular process” (GO:0009987), and “Single-organism process” (GO:0044699), which were assigned to 1,008, 959, and 810 DEGs, respectively. In the cellular component category, the most represented GO terms were “Cell” (GO:0005623), “Cell part” (GO:0044464), and “Membrane” (GO:0016020), which were assigned to 433, 433, and 412 DEGs, respectively. The most common molecular function GO terms were “Catalytic activity” (GO:0003824), “Binding” (GO:0005488), and “Transporter activity” (GO:0005215), which were assigned to 1,144, 672, and 136 DEGs, respectively. A total of 128 KEGG pathways were enriched among 1,457 DEGs. The main pathways were “Metabolic pathways” (ko01100), “Biosynthesis of secondary metabolites” (ko01110), and “Plant–pathogen interaction” (ko04626), which were associated with 649, 374, and 118 DEGs, respectively (Figure 6C).

### Metabolomic Analysis

#### Multivariate Statistical Analysis of the Metabolome

**FIGURE 7:**
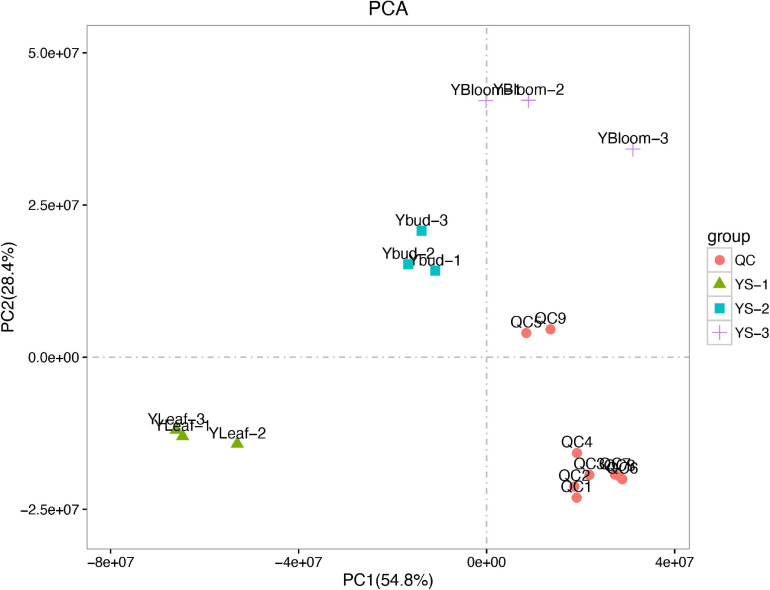
Principal component analysis of test and quality control samples.

A principal component analysis (PCA) can generally reflect the overall metabolic differences between samples in each group and the degree of variability between samples within a group. The first and second principal components (PC1 and PC2, respectively) of the sample group data for the *C. dichrum* leaves, flower buds, and blooming flowers accounted for 54.8 and 28.4% of the total variability, respectively (Figure 7). These results reflect relatively large overall metabolic differences, but low diversity between samples.

Qualitative and quantitative data were obtained for 568 metabolites in *C. dichrum* leaves, flower buds, and blooming flowers. Additionally, 216 annotated substances were highly abundant in the three plant tissues, including m-cresol, phytosphingosine, tartaric acid, abietic acid, neohesperidin, oxalic acid, salicylic acid, xylitol, glutamine, asparagine, 1-kestose, and other heterochromia-related metabolites. These compounds have diverse uses in medicines, skin care products, and food additives, indicative of the importance and utility of *C. dichrum*.

**FIGURE 8:**
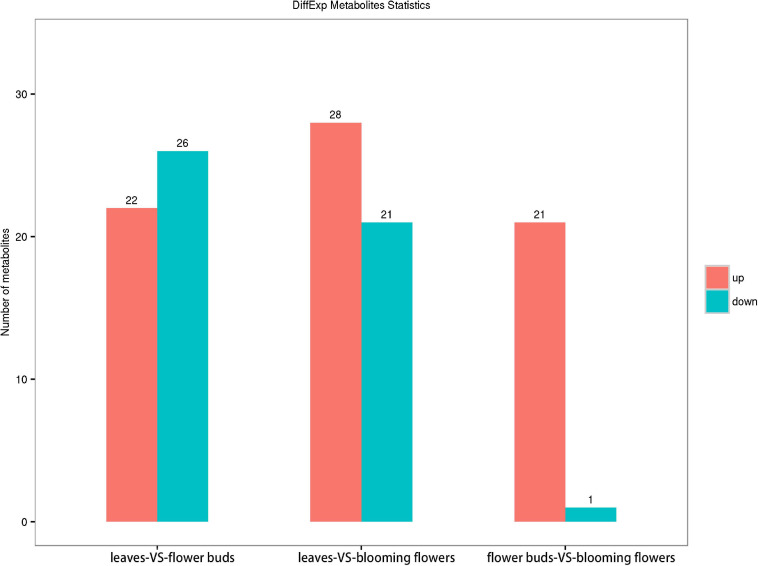
Comparison of metabolite contents among *Chrysanthemum dichrum* plant tissues.

We screened 48 metabolites with significant differences between the *C. dichrum* leaves and flower buds. Of these metabolites, 22 and 26 were, respectively, more and less abundant in flower buds than in leaves. A total of 49 metabolites differed significantly between the leaves and blooming flowers, of which 28 and 21 metabolites were respectively more and less abundant in blooming flowers than in leaves. A comparison between the flower buds and blooming flowers revealed 22 significantly different metabolites, with all but one more abundant in the blooming flowers than in the flower buds (Figure 8).

#### Clustering Analysis of Differentially Abundant Metabolites

**FIGURE 9:**
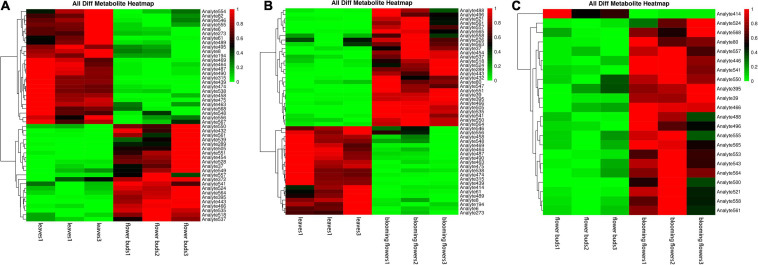
Clustering heat maps of differentially abundant metabolites among *Chrysanthemum dichrum* plant tissues. The abscissa is the sample of each period, and the ordinate on the right is the name of the metabolite. The redder part of the heat map indicates the higher the metabolite content. **(A)** Is the differential metabolite clustering heat map of leaves and flower buds, and **(B)** is the differential metabolite clustering heat map of leaves and blooming flowers comparison group. **(C)** Is the heat map of different metabolites clustering between flower buds and blooming flowers comparison group. The abscissa is the sample of each period, and the ordinate on the right is the name of the metabolite. The redder part of the heat map indicates the higher the metabolite content.

We compared the three tissue samples in groups, normalized the data for the differentially abundant metabolites, and conducted a cluster analysis. The following metabolites were more abundant in leaves than in flower buds: aminooxyacetic acid, alpha-ketoglutaric acid, melezitose, cellobiose, DL-dihydrosphingosine, galactinol, lactulose, conduritol, D-glyceric acid, glycerol, 2-hydroxypyridine, threonic acid, 1-kestose, proline, alanine, and palmitic acid. The opposite pattern was detected for the following metabolites: raffinose, isopropyl-beta-D-thiogalactopyranoside, methylmalonic acid, 3-cyanoalanine, glucose, methyl, phosphate, L-malic acid, glutamine, threonine, fumaric acid, oxoproline, asparagine, xylose, isoleucine, and aspartic acid (Figure 9A).

The following metabolites were more abundant in blooming flowers than in leaves: raffinose, isopropyl-beta-D-thiogalactopyranoside, leucine, lysine, methylmalonic acid, 3-cyanoalanine, phenylalanine, glucose, ribose, phosphate, L-malic acid, threonine, asparagine, oxoproline, xylose, serine, isoleucine, ethanolamine, aspartic acid, and valine. The opposite pattern was detected for the following metabolites: aminooxyacetic, alpha-ketoglutaric, melezitose, cellobiose, DL-dihydrosphingosine, galactinol, lactulose, D-glyceric, glycerol, and proline (Figure 9B).

Only one metabolite was more abundant in flower buds than in blooming flowers, but it was not identified. However, the following metabolites were determined to be more abundant in blooming flowers than in flower buds: 3,6-anhydro-D-galactose, isopropyl-beta-D-thiogalactopyranoside, leucine, lysine, gluconic acid, phenylalanine, glucose, threonine, glutamic acid, oxoproline, lactic acid, 1-kestose, xylose, serine, isoleucine, aspartic acid, valine, and palmitic acid (Figure 9C).

#### KEGG Annotation and KO Enrichment Analysis

**FIGURE 10:**
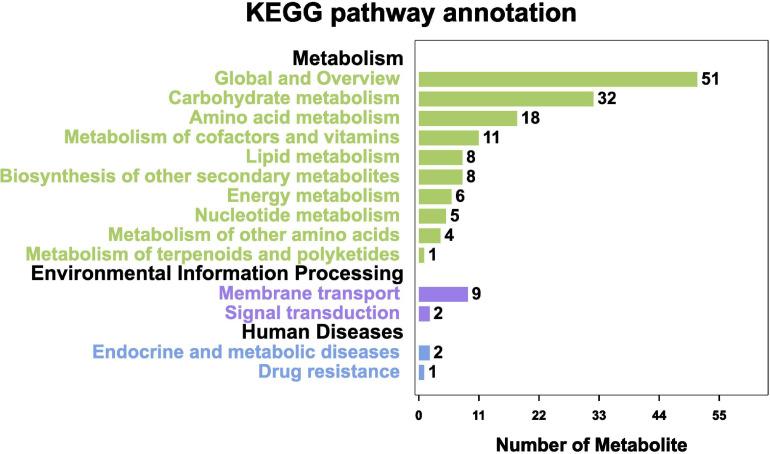
KEGG pathway annotation of metabolites.

On the basis of the KEGG pathway enrichment analysis, 296 metabolites were assigned to 17 metabolic pathways. The most represented pathway was the “Global and overview” (51 metabolites), followed by “Carbohydrate metabolism” (32 metabolites) and “Amino acid metabolism” (18 metabolites). The least annotated metabolic pathway was “Metabolism of terpenoids and polyketides” (1 metabolite) (Figure 10).

**FIGURE 11:**
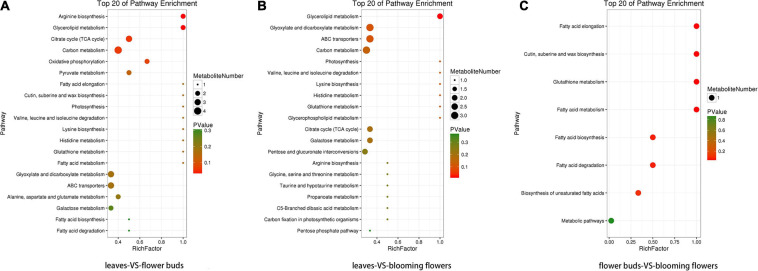
Enriched KO pathway bubble diagrams. **(A)** Shows the enrichment of KO pathways in leaves and flower buds, and **(B)** shows the main KO pathways in which metabolites in leaves and blooming flowers are enriched. **(C)** Shows the enrichment of KO pathway in flower buds and blooming flowers. The top 20 Pathways with the smallest *P* value (or *Q* value) were used for mapping. The ordinate was Pathway, and the abscissa was enrichment factor (the number of differences in this pathway divided by all numbers). The size represented the number, and the redder the color, the smaller the *P/Q* value.

The four KO pathways with the most differentially abundant metabolites and the smallest *P/Q* value between the leaves and flower buds were arginine biosynthesis, glycerolipid metabolism, citrate cycle (TCA cycle), and carbon metabolism (Figure 11A). Additionally, the four KO pathways with the most differentially abundant metabolites and the smallest *P*/*Q* value between the leaves and blooming flowers were glycerolipid metabolism, glyoxylate and dicarboxylate metabolism, ABC transporters, and carbon metabolism (Figure 11B). Furthermore, the four KO pathways with the most differentially abundant metabolites and the smallest *P/Q* value between the flower buds and blooming flowers were fatty acid elongation; cutin, suberine, and wax biosynthesis; glutathione metabolism; and fatty acid metabolism (Figure 11C).

#### Verification of Gene Expression Profiles Using qRT-PCR

To further verify the expression profiles of genes in the Illumina sequencing analyses, 15 ungenes with high expression levels and large differential multiple were selected for qRT-PCR, and leaf (YS1), flower bud (YS2) and blooming flower (YS3) were selected for RNA-seq. According to transcriptome sequencing data, the expression level of unigenes in the former group was significantly higher than that in the latter group in the three control groups. The RT-PCR results showed that the expression patterns of these 15 genes were consistent with the sequencing data (Additional file 10, Figure 12).

**FIGURE 12:**
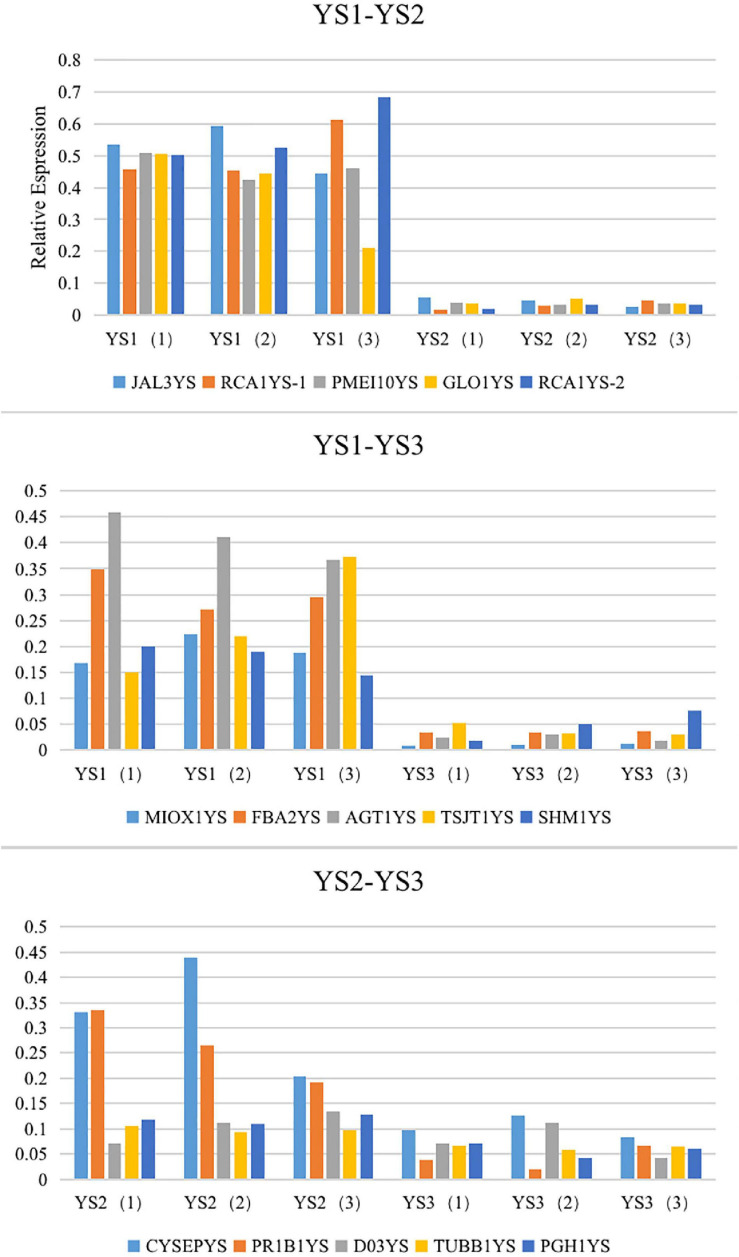
The expression profiles of 15 transcripts in *C. dichrum* by qRT-PCR.

### Conjoint Analyses of Transcriptome and Metabolome Sequencing

#### Common Metabolic Pathway Analysis

Due to the few types of differential metabolites among sample groups, and the absence of target metabolites in some differential metabolites, the common pathway analysis of all genes and all metabolites was carried out.

Combined analysis of transcriptome and metabolome sequencing showed that common pathway analysis was conducted for all genes and all metabolites. Due to the small number of differential metabolites among sample groups and the lack of target metabolites in some differential metabolites, we conducted common pathway analysis for all genes and metabolites.

There were 64 metabolic pathways shared by all genes and metabolites, 9016 candidate genes with pathway annotations, and 64 metabolites with pathway annotations. A total of 3,485 candidate genes (38.65%) and 42 metabolites (65.63%) were annotated for Metabolic pathways. A total of 1,917 candidate genes were annotated by Biosynthesis of secondary metabolites pathway, accounting for 21.26% of the total, and 18 metabolites were annotated, accounting for 28.13% of the total. A total of 570 candidate genes were annotated by Carbon Metabolism Pathway, accounting for 6.32% of the total, and 10 metabolites were annotated, accounting for 15.63% of the total. These three paths are the most annotated (Additional File 14).

#### Correlation Coefficient Model Analysis of Transcriptome and Metabolome

**FIGURE 13:**
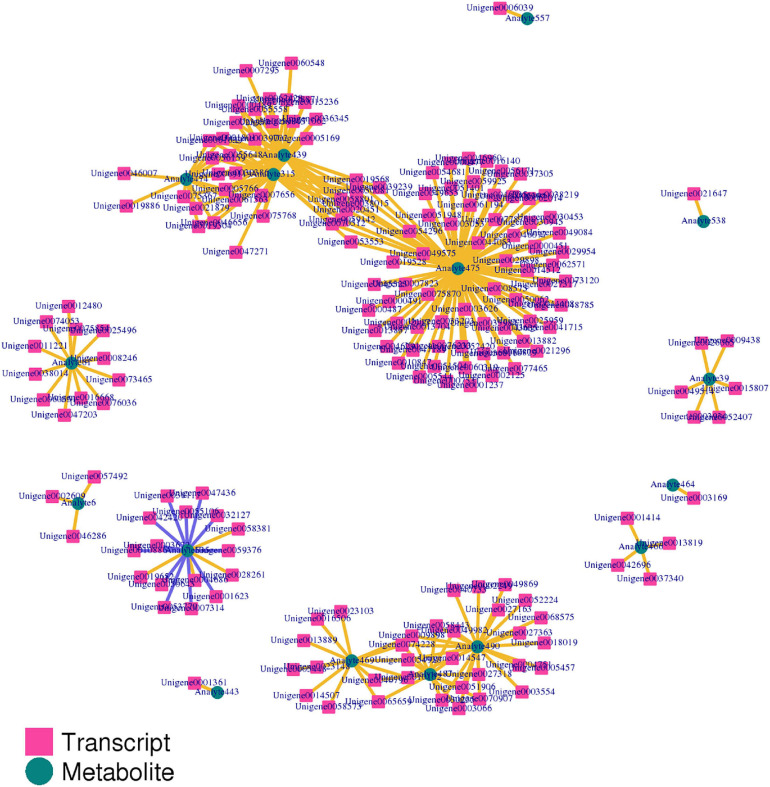
The correlation network diagram of gene expression and metabolite abundance.

The correlation between genes and metabolites was evaluated according to Pearson’s correlation coefficient. The top 250 differential genes and metabolites with the absolute correlation coefficient greater than 0.5 were screened out, and the correlation network diagram of gene expression and metabolite abundance was drawn (Figure 13). The results showed that there were more positive correlations between different genes and different metabolites, and only 12 groups showed negative correlations.

## Discussion

On the basis of a metabolomic analysis of *C. dichrum* tissue samples from three different developmental periods, we identified several differentially abundant functional metabolites among the 568 total metabolites. Two metabolites associated with food additives, raffinose and 1-kestose, were detected in leaves.

Raffinose, which is also called melitriose, is a trisaccharide comprising galactose, fructose, and glucose. Melitriose is a functional oligosaccharide that can induce substantial bifidobacterial proliferation. It can simultaneously promote the reproduction and growth of beneficial bacteria, such as bifidobacteria and lactobacilli, while also inhibiting the reproduction of harmful bacteria in the intestines, ultimately helping to establish a healthy intestinal microbial flora (Cox, 1966; Mussatto and Mancilha, 2007). Previous studies confirmed that melitriose can also prevent constipation and inhibit diarrhea; function as a two-way regulator to detoxify and protect the liver; inhibit the production of toxins in the body; minimize the burden on the liver; regulate the human immune system; enhance immunity; decrease allergic reactions following an oral administration (Zhang et al., 2004, 2008; Zhang R. et al., 2013). It also can improve lipid metabolism, lower blood lipid and cholesterol levels, and prevent dental caries; however, it is not effective against oral cariogenic bacteria. Additionally, even in the presence of sucrose, melitriose can decrease tartar formation and protect regions where oral microorganisms are deposited and produce acid, thereby preventing corrosion. It can also whiten and strengthen teeth. Because melitriose is a low-calorie trisaccharide, it does not affect the blood sugar level in the human body and can be consumed by people with diabetes (Yazawa and Tamura, 1982; Muthukumaran et al., 2018).

As the smallest oligofructose (i.e., fructooligosaccharide) compound, 1-kestose is an active ingredient naturally found in diverse foods, including fruits, vegetables, and honey, and it is an excellent water-soluble dietary fiber. Fructooligosaccharides, which comprise 1-kestose (GF2), nystose (GF3), and 1F-fructofruranosy 1 nystose (GF4) (Jayalakshmi et al., 2021), can activate bifidobacteria, while also regulating the intestinal microbial flora balance, ensuring the bowel is appropriately hydrated, regulating blood lipid levels, lowering cholesterol and blood sugar levels, and enhancing immunity (Li et al., 2004; Probert et al., 2004; Mabel et al., 2008; Hidaka et al., 2010; Delgado et al., 2012). Owing to its superior physiological functions, oligofructose has become a widely popular functional food component in the international food market during the past 10 years. It has been used in more than 500 food and health products and medicines. Moreover, it has been described as a healthy sugar for the 21st century (Yun, 1996).

Metabolites (e.g., glutamine) in edible medicinal plants have been detected in *C. dichrum* flower buds. Basic and clinical experiments demonstrated that glutamine can decrease catabolic activities, promote protein synthesis, improve immune functions, protect the intestinal mucosal barrier, and accelerate wound healing. It can also be used to improve gastrointestinal health by preventing gastric and duodenal ulcers, gastritis, and hyperacidity (Taxuke et al., 2003). Asparagine is a medically relevant metabolite that can lower blood pressure, expand bronchial tubes (useful for treating asthmatics), and prevent peptic ulcers and gastric dysfunction. It can also be used for cultivating microorganisms and treating acrylonitrile wastewater.

In this study, raffinose and asparagine contents were higher in *C. dichrum* blooming flowers than in leaves. Additionally, another metabolite, xylose, was also highly abundant in blooming flowers. This sugar has been used as a food additive. As a component of xylan, it is widely distributed in plants. Xylose is not digested and absorbed, making it useful as a sweetener that will not lead to weight gain. It can also activate and promote the growth of bifidobacteria in the human intestine, with beneficial effects on health. Ingesting foods containing xylose can improve the microbial environment of the human body and enhance immunity. It cannot be used by microorganisms in the oral cavity. Additionally, it has some physiological functions of dietary fiber and it can decrease serum cholesterol levels and prevent colon cancer. Hence, adding a small amount of xylose to food can lead to increased positive health effects (Liang and Zhao, 2011). Xylose is widely present in plant hemicellulose as macromolecular xylan and can be obtained by degrading xylan with acids or enzymes (Harner et al., 2015). Xylooligosaccharides are an important component of functional foods. Because they are stable and non-toxic sugars that can be efficiently purified, xylooligosaccharides are increasingly being used in food, feed, and medicine, while also being applied in other fields (Zhang Y.H. et al., 2013; Baker et al., 2021).

In the current study, the 1-kestose and xylose contents were high in blooming flowers. Similarly, many other functional metabolites were more abundant in blooming flowers than in flower buds and leaves, including tartaric acid and salicylic acid. Tartaric acid is an antioxidant that has been added to foods. It is most commonly used as a beverage additive and a raw material for the pharmaceutical industry (Kroschwitz and Seidel, 2004). Earlier research proved that salicylic acid is useful for treating acne and for lightening post-acne pigmentation, minimizing pore size, removing fine wrinkles, and preventing sun-induced skin aging. The renewal of skin cells in response to salicylic acid will result in smoother skin (Kligman and Kligman, 1998). In small amounts, salicylic acid is also applicable as a food preservative (Chrubasik et al., 2000). It is also an important raw material for the pharmaceutical industry.

The data presented herein indicate that *C. dichrum* flowers and leaves are rich in metabolites with important functions. Consequently, *C. dichrum* is an important plant source of useful compounds. In terms of the extraction of functional metabolites from *C. dichrum*, the flowers at full bloom are likely the most appropriate plant tissues because they contain large amounts of diverse metabolites.

## Materials and Methods

### Plant Materials and RNA Extraction

The *C. dichrum* plants used in this study were cultivated at 23°C in a greenhouse at the Beijing Academy of Agriculture and Forestry Sciences (116.3°E, 39.9°N) with an 8-h light/16-h dark cycle. Leaves, flower buds, and blooming flowers were collected from *C. dichrum* plants, with three biological replicates per sample. This plant samples was used for both transcriptome analysis and Metabolomic analysis. The collected samples were quickly frozen in liquid nitrogen and stored at −80°C. The RNeasy Plant Mini Kit (Qiagen, China) was used to extract total RNA from the frozen samples. The concentration of the RNA was determined using the NanoDrop ND2000 spectrophotometer (Thermo Scientific).

### Library Construction and Sequencing

To construct a sequencing library, rRNA was eliminated from the total RNA extracted from the leaves, flower buds, and blooming flowers using the Ribo-Zero^TM^ Magnetic Kit (Epicenter), after which the mRNA was enriched using oligo-(dT) beads. Fragmentation buffer was used to produce short mRNA fragments, which were then reverse transcribed into cDNA. The second cDNA strand was synthesized in buffer containing DNA polymerase I, RNase H, and dNTP. The cDNA fragments were purified using the QiaQuick PCR extraction kit. After repairing the ends and adding a poly(A) tag, an Illumina sequencing adapter was ligated. The size of the ligation products was determined by Gene *de novo* using the Illumina HiSeq^TM^ 4000 system (Illumina, San Diego, CA, United States) for the subsequent agarose gel electrophoresis, PCR amplification, and sequencing. The PacBio Sequel system (PacBio, CA, United States) was used for sequencing. To improve the accuracy of the PacBio data, the reads were filtered by deleting reads with adapters, reads with more than 10% unknown nucleotides (N), and reads with more than 40% low-quality nucleotides (*Q* value ≤ 20). The CD-HIT (version 4.6.7) software (with a sequence identity threshold of 0.99) was used to remove redundant sequences to obtain the final unigene sequences.

### Basic Annotation of Unigenes

Unigenes were annotated using the BLASTX algorithm and the following four databases (E value of 1.00E-5): Nr (NCBI), Swiss-Prot^1^, KEGG^2^, and KOG^3^. The sequence direction of each gene was determined according to the best alignments. Unigenes were annotated with GO terms using the Blast2GO program (Conesa et al., 2005). On the basis of the Blast2GO analysis, we selected high-quality unigenes with a high hit rate. These unigenes were functionally classified using the WEGO software (Ye, 2006).

### Analysis of *Chrysanthemum dichrum* Transcriptome Sequencing Results

The RPKM values derived from the RNA-seq data were used to represent unigene expression levels (Fourquin et al., 2005). The DEGs in the chrysanthemum transcriptome were screened and analyzed as previously described (Samarskiǐ and Claverie, 1997). To verify the *P*-value threshold, the FDR was used in multiple tests and analyses (Endress, 2006). The threshold for determining a significant difference in gene expression was set as follows: absolute value of the log_2_(ratio) ≥ 2 and FDR < 0.05 (Mortazavi et al., 2008). Differential gene expression was analyzed for the DEGs with expression levels that differed by at least 2-times between samples.

### Alternative Splicing Detection

To analyze alternative splicing events in a unigene, transcripts were divided into gene families according to k-mer similarity. This process uses the coding genome reconstruction tool (Cogent) and then recombines each family into a coding reference genome. This process uses the De Bruijn diagram (Li et al., 2017). The SUPPA tool was used to analyze the alternative splicing events of a unigene (Alamancos et al., 2015).

### Gene Expression Analysis Based on qRT-PCR

To conduct qRT-PCR assays, the total RNA extracted from the leaves, flower buds, and blooming flowers were treated with DNase (Promega, United States) to eliminate any residual genomic DNA. The purified RNA served as the template for synthesizing cDNA using a commercial reverse transcription kit (Tsingke, China). The qRT-PCR analysis was completed using the PikoReal system (Thermo Fisher Scientific, Germany). The 20-μL reaction solution comprised 1 μL reverse transcribed cDNA as the template. The PCR program was as follows: 95°C for 30 s; 40 cycles of 95°C for 5 s and 60°C for 30 s. Details regarding the qRT-PCR primers used to determine the relative expression levels of specific genes are listed in Additional file 13. Each sample was analyzed in triplicate, with three biological replicates. The 2^–ΔΔ*Ct*^ method was used to calculate relative gene expression levels. The reference control was the *Aspergillus* gene encoding protein phosphatase 2A (PP2Ac) (Xue et al., 2014).

### Chemicals and Reagents

The chemicals and reagents used in this study, such as methanol, ethanol, and acetonitrile, were purchased from Merck (Germany;^4^). The Milli-Q system (Millipore, Bedford, MA, United States) was used to produce ultrapure water. Authentic standards were purchased from BioBioPha Co., Ltd.^5^ and Sigma-Aldrich^6^. All chemicals and reagents were of analytical grade.

### Sample Preparation and Extraction

Freeze-dried samples were ground to a powder using zirconia beads and a mixing mill (MM 400, Retsch) set at 30 Hz for 1.5 min. The powdered material was weighed, after which 100 mg was mixed with 1.0 mL 70% methanol aqueous solution (containing 0.1 mg/L lidocaine as an internal standard) overnight at 4°C. After centrifuging the mixture at 10,000 *g* for 10 min, the supernatant was collected and filtered (SCAA-104; pore size 0.22 μm; ANPEL, Shanghai, China;^7^) for the subsequent LC-MS/MS analysis. To assess the repeatability of the entire experiment, quality control samples were mixed with all samples.

### AB Sciex QTRAP6500 (UPLC) Analysis

The extracted compounds were analyzed using an LC-ESI-MS/MS system [UPLC: Shim-pack UFLC SHIMADZU CBM30A^8^; MS/MS: Applied Biosystems 6500 QTRAP^9^] (Chen W. et al., 2013; Wishart et al., 2013). For each sample, a 2-μL aliquot was injected into the Waters ACQUITY UPLC HSS T3 C18 column (2.1 mm × 100 mm, 1.8 μm) operating at 40°C with a flow rate of 0.4 mL/min. The mobile phases used were acidified water (0.04 % acetic acid) (Phase A) and acidified acetonitrile (0.04 % acetic acid) (Phase B). Compounds were separated using the following gradient: 95:5 Phase A/Phase B at 0 min; 5:95 Phase A/Phase B at 11.0 min; 5:95 Phase A/Phase B at 12.0 min; 95:5 Phase A/Phase B at 12.1 min; and 95:5 Phase A/Phase B at 15.0 min. The eluent was analyzed by an ESI-triple quadrupole-linear ion trap (QTRAP) mass spectrometer. The LIT and triple quadrupole (QQQ) scans were acquired using a triple quadrupole-linear ion trap mass spectrometer (QTRAP; AB Sciex QTRAP6500 System) equipped with an ESI-Turbo Ion-Spray interface. The system was operated in the positive ion mode and controlled with the Analyst 1.6.1 software (AB Sciex). The operating parameters were as follows: ESI source temperature, 500°C; ion spray voltage, 5,500 V; curtain gas, 25 psi; and collision-activated dissociation, highest setting. The QQQ scans were acquired as MRM experiments with optimized declustering potential and collision energy for each MRM transition. The m/z range was set between 50 and 1,000.

### Data Preprocessing and Metabolite Identification

To generate a matrix containing relatively little offset and redundant data, the peak signal/noise was manually checked (>10) and an internal Perl software was used to remove redundant signals resulting from different isotopes and to analyze intra-source fragmentation and K^+^, Na^+^, and NH_4_^+^ adducts and dimers. The accurate m/z of each Q1 was obtained, after which the area of each chromatographic peak was calculated. The peaks for the different samples were aligned according to the spectrum and retention time. Metabolites were identified by searching internal and public databases [MassBank, KNApSAcK, HMDB (Zhu et al., 2013), MoTo DB, and METLIN (Worley and Powers, 2013)]. The m/z values, RT, and lysis patterns were compared with standards. The data filtering as well as the peak detection, comparison, and calculation were performed using the Analyst 1.6.1 software.

### Multivariate Statistical Analysis

For all samples, the R package model^10^ was used to perform the unsupervised dimensionality reduction PCA to initially visualize the differences between different sample groups (Kanehisa et al., 2008).

### Differentially Abundant Metabolite Analysis

The *T* test and the variable importance in the projection (VIP) score of the (O)PLS model were used to rank the metabolites with significant differences in abundance between two groups. The *P*-value threshold for the *T* test was set at < 0.05 and the VIP score threshold was set at ≥ 1.

### KEGG Pathway Analysis

The enriched KEGG metabolic pathways among the metabolites were determined. The metabolic and signal transduction pathways significantly enriched among the differentially abundant metabolites were identified using the following formula:

$$P=1-\sum_{i=0}^{m-1}\frac{\left(\begin{array}{c} M\\ i \end{array}\right)\left(\begin{array}{c} N-M\\ n-i \end{array}\right)}{\left(\begin{array}{c} N\\ n \end{array}\right)}$$

where N is the number of all metabolites annotated using the KEGG database, n is the number of different metabolites in N, M is the number of all metabolites assigned to a specific pathway, and m is the number of different metabolites in M. The *P*-value was adjusted using an FDR ≤ 0.05. The pathways satisfying this condition were identified as significantly enriched among the differentially abundant metabolites.

### Transcriptome Data

The Submission ID: SUB9976541.

The BioProject ID: PRJNA744998.

Access link: https://www.ncbi.nlm.nih.gov/sra/PRJNA744998.

## Data Availability Statement

The datasets presented in this study can be found in online repositories. The names of the repository/repositories and accession number(s) can be found in NCBI, accession number PRJNA744998.

## Author Contributions

HL, XiaC, HC, and JL performed the research. XiaC analyzed the data and prepared the manuscript. CH and YJ guided the research. CL, DC, and XiC provided assistance for the research. All authors read and approved the final manuscript.

## Conflict of Interest

The authors declare that the research was conducted in the absence of any commercial or financial relationships that could be construed as a potential conflict of interest.

## Publisher’s Note

All claims expressed in this article are solely those of the authors and do not necessarily represent those of their affiliated organizations, or those of the publisher, the editors and the reviewers. Any product that may be evaluated in this article, or claim that may be made by its manufacturer, is not guaranteed or endorsed by the publisher.

## Footnotes

1
http://www.expasy.ch/sprot

2
http://www.genome.jp/kegg

3
http://www.ncbi.nlm.nih.gov/KOG

4
www.merckchemicals.com

5
www.biobiopha.com/

6
www.sigmaaldrich.com/unitedStates.html

7
www.anpel.com.cn/

8
http://www.shimadzu.com.cn/

9
http://www.appliedbiosystems.com.cn/

10
http://www.r-project.org/

## Supplementary Material

Supplementary Table 1 Genes differentially expressed between *Chrysanthemum dichrum* leaves and flower buds.

Supplementary Table 2 Genes differentially expressed between *Chrysanthemum dichrum* leaves and blooming flowers.

Supplementary Table 3 Genes differentially expressed between *Chrysanthemum dichrum* flower buds and blooming flowers.

Supplementary Table 4 GO functional categorization of significantly up-regulated and down-regulated differentially expressed genes between *Chrysanthemum dichrum* leaves and flower buds.

Supplementary Table 5 Enriched KEGG pathways among the significantly up-regulated and down-regulated differentially expressed genes between *Chrysanthemum dichrum* leaves and flower buds.

Supplementary Table 6 GO functional categorization of significantly down-regulated differentially expressed genes between *Chrysanthemum dichrum* leaves and flower buds.

Supplementary Table 7 Enriched KEGG pathways among the significantly up-regulated and down-regulated differentially expressed genes between *Chrysanthemum dichrum* leaves and blooming flowers.

Supplementary Table 8 GO functional categorization of significantly up-regulated and down-regulated differentially expressed genes between *Chrysanthemum dichrum* flower buds and blooming flowers.

Supplementary Table 9 Enriched KEGG pathways among the significantly up-regulated differentially expressed genes between *Chrysanthemum dichrum* leaves and blooming flowers.

Supplementary Table 10 Details regarding the 15 primers used for the quantitative real-time PCR analysis of *Chrysanthemum dichrum* genes.

Supplementary Table 11 Enriched KEGG pathways among the significantly down-regulated differentially expressed genes between *Chrysanthemum dichrum* leaves and blooming flowers.

Supplementary Table 12 Significantly different metabolites between *Chrysanthemum dichrum* leaves and blooming flowers.

Supplementary Table 13 Significantly different metabolites between *Chrysanthemum dichrum* flower buds and blooming flowers.

Supplementary Table 14 Annotation of metabolic pathways common to all genes and metabolites.

## References

[B1] Alamancos G. P. Pagès A. Trincado J. L. Bellora N. Eyras E. (2015). Leveraging transcript quantification for fast computation of alternative splicing profiles. *RNA* 21 1521–1531. 10.1261/rna.051557.115 26179515 PMC4536314

[B2] Baker J. T. Duarte M. E. Holanda D. M. Kim S. W. (2021). Friend or foe impacts of dietary xylans, xylooligosaccharides, and xylanases on intestinal health and growth performance of monogastric animals. *Animals* 11:609. 10.3390/ani11030609 33652614 PMC7996850

[B3] Chen L. Chen Y. Jiang J. Chen S. Chen F. Guan Z. (2012). The constitutive expression of *Chrysanthemum dichrum* ICE1 in *Chrysanthemum grandiflorum* improves the level of low temperature, salinity and drought tolerance. *Plant Cell Rep.* 31 1747–1758. 10.1007/s00299-012-1288-y 22645020

[B4] Chen S. Miao H. Chen F. Jiang B. Lu J. Fang W. (2009). Analysis of expressed sequence tags (ESTs) collected from the inflorescence of *Chrysanthemum*. *Plant Mol. Biol. Rep.* 27 503–510. 10.1007/s11105-009-0103-6

[B5] Chen W. Gong L. Guo Z. Wang W. Zhang H. Liu X. (2013). A novel integrated method for large-scale detection, identification, and quantification of widely targeted metabolites: application in the study of rice metabolomics. *Mol. Plant* 6 1769–1780. 10.1093/mp/sst080 23702596

[B6] Chen Y. Chen S. Chen F. Li P. Chen L. Guan Z. (2012). Functional characterization of a *Chrysanthemum dichrum* stress-related promoter. *Mol. Biotechnol.* 52 161–169. 10.1007/s12033-011-9483-6 22187168

[B7] Chen Y. Jiang J. Song A. Chen S. Shan H. Luo H. (2013). Ambient temperature enhanced freezing tolerance of *Chrysanthemum dichrum* CdICE1 *Arabidopsis* via miR398. *BMC Biol.* 11:121. 10.1186/1741-7007-11-121 24350981 PMC3895800

[B8] Chi T. Xu T. Liu Y. Ma J. Guan Z. Fang W. (2018). Genetic variation for cold tolerance in an interspecific *C. dichrum* × *C. nankingense* population. *J. Nuclear Agric. Sci.* 32 2298–2304.

[B9] Chrubasik S. Eisenberg E. Balan E. Weinberger T. Luzzati R. Conradt C. (2000). Treatment of low back pain exacerbations with willow bark extract:a randomized double-blind study. *Am. J. Chin. Med.* 109 9–14. 10.1016/s0002-9343(00)00442-3 10936472

[B10] Conesa A. Götz S. García-Gómez J. M. Terol J. Talón M. Robles M. (2005). Blast2GO: a universal tool for annotation, visualization and analysis in functional genomics research. *Bioinformatics* 21 3674–3676. 10.1093/bioinformatics/bti610 16081474

[B11] Cox C. S. (1966). The survival of *Escherichia coli* sprayed into air and into nitrogen from distilled water and from solutions of protecting agents, as a function of relative humidity. *J. Gen. Microbiol.* 43 383–399. 10.1099/00221287-43-3-383 5336475

[B12] Delgado G. T. Thomé R. Gabriel D. L. Tamashiro W. M. Pastore G. M. (2012). Yacon(*Smallanthus sonchifolius*)-derived fructooligosaccharides improves the immune parameters in the mouse. *Nutr. Res.* 32 884–892. 10.1016/j.nutres.2012.09.012 23176799

[B13] Endress P. K. (2006). Angiosperm floral evolution: morphological developmental framework. *Adv. Bot. Res.* 44 1–61. 10.1016/s0065-2296(06)44001-5

[B14] Fourquin C. Vinauger-Douard M. Fogliani B. Dumas C. Scutt C. (2005). Evidence that CRABS CLAW and TOUSLED have conserved their roles in carpel development since the ancestor of the extant angiosperms. *Proc. Natl. Acad. Sci. U.S.A.* 102 4649–4654. 10.1073/pnas.0409577102 15767586 PMC555504

[B15] Harner N. K. Wen X. Bajwa P. K. Austin G. D. Ho C. Y. Habash M. B. (2015). Genetic improvement of native xylose-fermenting yeasts for ethanol production. *J. Ind. Microbiol. Biotechnol.* 42 1–20. 10.1007/s10295-014-1535-z 25404205

[B16] Hidaka H. Eida T. Takizawa T. Tokunaga T. Tashiro Y. (2010). Effects of fructooligosaccharides on intestinal flora and human health. *Bifidobacteria Microflora* 5 37–50. 10.12938/bifidus1982.5.1_37

[B17] Hong Y. Tang X. Huang H. Zhang Y. Dai S. (2015). Transcriptomic analyses reveal species-specific light-induced anthocyanin biosynthesis in *Chrysanthemum*. *BMC Genomics* 16:202. 10.1186/s12864-015-1428-1 25887322 PMC4404602

[B18] Hsianshiu K. (1993). *Flora Republicae Popularis Sinicae (FRPS)*, Vol. 76. Beijing: Science Press, 47.

[B19] Jayalakshmi J. Mohamed Sadiqa A. Sivakumarb V. (2021). Microbial enzymatic production of fructooligosaccharides from sucrose in agricultural harvest. *Asian J. Microbiol. Biotechnol. Environ. Sci.* 23 84–88.

[B20] Kanehisa M. Araki M. Goto S. Hattori M. Hirakawa M. Itoh M. (2008). KEGG for linking genomes to life and the environment. *Nucleic Acids Res.* 36 D480–D484. 18077471 10.1093/nar/gkm882 PMC2238879

[B21] Kligman D. Kligman A. M. (1998). Salicylic acid peels for the treatment of photoaging. *Dermatol. Surg.* 24 325–328. 10.1111/j.1524-4725.1998.tb04162.x 9537006

[B22] Kroschwitz J. Seidel A. (2004). *Kirk-Othmer Encyclopedia of Chemical Technology. CRC Handbook*, 5 Edn. Hoboken, NJ: Wiley-Interscience.

[B23] Li J. Harata-Lee Y. Denton M. D. Feng Q. Rathjen J. R. Qu Z. (2017). Long read reference genome-free reconstruction of a full-length transcriptome from *Astragalus membranaceus* reveals transcript variants involved in bioactive compound biosynthesis. *Cell Discov.* 3:17031. 10.1038/celldisc.2017.31 PMC5573880 28861277

[B24] Li J. Wang J. Kaneko T. Qin L. Q. Sato A. (2004). Effects of fiber intake on the blood pressure, lipids, and heart rate in Goto Kakizaki rats. *Nutrition* 20 1003–1007. 10.1016/j.nut.2004.08.010 15561491

[B25] Li P. Chi T. Liu Y. Fan H. Wang H. Guan Z. (2020). Genetic polymorphism analysis, evaluation of drought tolerance and association analysis using SSR markers in the interspecific *Chrysanthemum dichrum*×*C.nankingense* F1hybrids. *J. Nanjing Agric. Univ.* 43 238–246.

[B26] Liang C. S. Zhao G. Z. (2011). Terahertz spectroscopic inspection and analysis of Xylitol and D-Xylose. *Spectrosc. Spectr. Anal.* 31 323–327. 21510372

[B27] Liu H. Sun M. Du D. Pan H. Cheng T. Wang J. (2015). Whole-transcriptome analysis of differentially expressed genes in the vegetative buds, floral buds and buds of *Chrysanthemum morifolium*. *PLoS One* 10:e0128009. 10.1371/journal.pone.0128009 26009891 PMC4444331

[B28] Liu Z. Ma L. Nan Z. Wang Y. (2013). Comparative transcriptional profiling provides insights into the evolution and development of the zygomorphic flower of *Vicia sativa* (Papilionoideae). *PLoS One* 8:e57338. 10.1371/journal.pone.0057338 23437373 PMC3578871

[B29] Mabel M. J. Sangeetha P. T. Kalpana P. Srinivasan K. Prapulla S. G. (2008). Physicochemical characterization of fructooligosaccharides and evaluation of their suitability as a potential sweetener for diabetics. *Carbohydr. Res.* 343 56–66. 10.1016/j.carres.2007.10.012 18005951

[B30] Mortazavi A. Williams B. A. McCue K. Schaeffer L. Wold B. (2008). Mapping and quantifying mammalian transcriptomes by RNA-Seq. *Nat. Methods* 5 621–628. 10.1038/nmeth.1226 18516045 PMC13303166

[B31] Mussatto S. I. Mancilha I. M. (2007). Non-digestible oligosaccharides: a review. *Carbohydr. Polym.* 68 587–597. 10.1016/j.carbpol.2006.12.011

[B32] Muthukumaran P. Thiyagarajan G. Babu R. A. Lakshmi B. S. (2018). Raffinose from *Costus speciosus* attenuates lipid synthesis through modulation of PPARs/SREBP1c and improves insulin sensitivity through PI3K/AKT. *Chem. Biol. Interact.* 284 80–89. 10.1016/j.cbi.2018.02.011 29458019

[B33] Probert H. M. Apajalahti J. H. Rautonen N. Stowell J. Gibson G. R. (2004). Polydeztrose, lactitol and fructo-ligosaccharide fermentation by colonic bacteria in a three stage continuous culture system. *Appl. Environ. Microbiol.* 70 4505–4516. 10.1128/aem.70.8.4505-4511.2004 15294779 PMC492322

[B34] Ren L. Sun J. Chen S. Gao J. Dong B. Liu Y. (2014). A transcriptomic analysis of *Chrysanthemum nankingense* provides insights into the basis of low temperature tolerance. *BMC Genomics* 15:844. 10.1186/1471-2164-15-844 25277256 PMC4197275

[B35] Samarskiǐ A. Claverie J. M. (1997). The significance of digital gene expression profiles. *Genome Res.* 7 986–995. 10.1101/gr.7.10.986 9331369

[B36] Taxuke Y. Wasa M. Shimizu Y. Wang H. S. Okada A. (2003). Alanyl-glulamine-supplement edparenteral nut nition prevents intesti nal islemia-reperfusion injury in rats. *J. Parenter. Enteral Nutr.* 27 110–115. 10.1177/0148607103027002110 12665166

[B37] Wang H. Jiang J. Chen S. Qi X. Peng H. Li P. (2013). Next-generation sequencing of the *Chrysanthemum nankingense* (Asteraceae) transcriptome permits large-scale unigene assembly and SSR marker discovery. *PLoS One* 8:e62293. 10.1371/journal.pone.0062293 23626799 PMC3633874

[B38] Wishart D. S. Jewison T. Guo A. C. Wilson M. Knox C. Liu Y. (2013). HMDB 3.0—the human metabolome database in 2013. *Nucleic Acids Res.* 41 D801–D807. 23161693 10.1093/nar/gks1065 PMC3531200

[B39] Worley B. Powers R. (2013). Multivariate analysis in metabolomics. *Curr. Metabolomics* 1 92–107. 10.2174/2213235x130108 26078916 PMC4465187

[B40] Xu Y. Gao S. Yang Y. Huang M. Cheng L. Wei Q. (2013). Transcriptome sequencing and whole genome expression profiling of chrysanthemum under dehydration stress. *BMC Genomics* 14:662. 10.1186/1471-2164-14-662 24074255 PMC3849779

[B41] Xue C. M. Xie T. N. Ye S. D. Chen C. (2014). Reference gene selection for quantitative real-time PCR in *Pandora neoaphidis*. *J. Agric. Biotechnol.* 22 1575–1583.

[B42] Yazawa K. Tamura Z. (1982). Search for;sugar sources for selective increase of bifido-bacteria. *Bifidobacteria Microfora* 1 39–44.

[B43] Ye J. (2006). WEGO: a web tool for plotting GO annotations. *Nucleic Acids Res.* 34 W293–W297. 16845012 10.1093/nar/gkl031 PMC1538768

[B44] Yun J. W. (1996). Fructooligosaccharides—occurrence, preparation and application. *Enzyme Microb. Technol.* 19 107–117. 10.1016/0141-0229(95)00188-3

[B45] Zhang R. Zhao Y. Sun Y. Lu X. Yang X. (2013). Isolation, characterization, and he-patoprotective effects of the raffinose family oligosaccharides from *Rehmannia glu-tinosa* Libosch. *J. Agric. Food Chem.* 61 7786–7793. 10.1021/jf4018492 23879777

[B46] Zhang R. Zhou J. Jia Z. Zhang Y. Gu G. (2004). Hypoglycemic effect of *Rehmannia glutinosa* oligosaccharide in hyperglycemic and alloxan-induced diabetic rats and its mechanism. *J. Ethnopharmacol.* 90 39–43. 10.1016/j.jep.2003.09.018 14698506

[B47] Zhang R. X. Li M. X. Jia Z. P. (2008). *Rehmannia glutinosa*: review of botany, chemistry and pharmacology. *J. Ethnopharmacol.* 117 199–214. 10.1016/j.jep.2008.02.018 18407446

[B48] Zhang Y. H. Zhou W. Li B. (2013). Determination of sugar alcohols sweeteners in sugar-free food by derivatization capillary gas chromatography. *Chin. J. Anal. Chem.* 41 911–916. 10.3724/sp.j.1096.2013.20897

[B49] Zhu Z. J. Schultz A. W. Wang J. Johnson C. H. Yannone S. M. Patti G. J. (2013). Liquid chromatography quadrupole time-of-flight mass spectrometry characterization of metabolites guided by the METLIN database. *Nat. Protoc.* 8 451–460. 10.1038/nprot.2013.004 23391889 PMC3666335

