# Supplementary material for: Transcriptome and Metabolome Analyses of the Flowers and Leaves of Chrysanthemum dichrum
Source: Front Genet. 2021 Aug 31;12:716163. doi: 10.3389/fgene.2021.716163 (PMC8438430; doi:10.3389/fgene.2021.716163)
Supplement: Supplementary Table 10 — Details regarding the 15 primers used for the quantitative real-time PCR analysis of Chrysanthemum dichrum genes. [file Table_10.DOCX]

Primers used in real-time quantitative PCR of *Chrysanthemum* *dichrum* genes

| Gene name | Forward primer sequence (5′-3′) | Reverse primer sequence (5′-3′) | Correlation between RNA-Seq and qRT-PCR (R2) |
| --- | --- | --- | --- |
| *JAL3YS* | TAACCACGGGCAGTGAGAA | GGCACCGATTGGGAAG | 1 |
| *RCA1YS-1* | TAAGTGTTACAAAGAACGACCC | AAGGACGCCATTCAGAGC | 1 |
| *PMEI10YS* | GTTTGACAACATACCCTACACTCT | GAGGAGGTGGTGCTGGT | 1 |
| *GLO1YS* | TCTTCTGAAAGGCTGTTGG | TACTATGCTTCGGGTGCTG | 1 |
| *RCA1YS-2* | GGGGAGCATAAAGTGTTGAG | GCAGGAAGAATGGGTGGA | 1 |
| *MIOX1YS* | GATGAGCATTTGGGAGTGTT | GCCAGTCTTGGTTAGGGTAG | 1 |
| *FBA2YS* | TGAGGCTAAACGCTTTCCAC | CCTGTTTCCGCAGTTCG | 1 |
| *AGT1YS* | ACGGTTACTGCGGTGGTT | TTGTTTAGCCCAAGACCC | 0.99 |
| *TSJT1YS* | TGTATTTGTCTCCCTTAGTGCT | AGCAAATGACTTGGAGCAG | 0.98 |
| *SHM1YS* | AGCCTTGTATTCTGCTGTTGT | TCAACCAGGCTGTCTTTCC | 0.99 |
| *CYSEPYS* | TTGAATCGGTTTGCTGACTT | CCGCAAATGCCCAAC | 0.99 |
| *PR1B1YS* | TGTTATGATTTCACGGCTCG | GTTGTTCCACAGGCTTCC | 0.99 |
| *D03YS* | TGCCACGGGCTCATCATAGA | CGTCTGGGCTAAGAGTGCTA | 0.99 |
| *TUBB1YS* | TTGATTCGGTTCTTGATGTT | AGGGCTCAACGACTGTATCA | 0.98 |
| *PGH1YS* | AGTGACTGCCCATTATTGTGA | GCAAACCTGTTGAACCCTACT | 1 |
| *cmClUBI3*（Reference gene） | AGCTGAGCAGACTCCCGATG | AGGCGAATCATCAGTACCAAGT |  |
